# Genomic and epidemiological evidence linking a pre-season dengue case to local overwintering transmission in Guangdong, China

**DOI:** 10.1371/journal.pntd.0014221

**Published:** 2026-08-21

**Authors:** Liling Lin, Man Liu, Jianqian Chen, Qihua Guan, Siyang Jiang, Xiaohua Tan, Xin Zhang, Penghui Jia, Jinhua Duan, Hui Deng, Qiulan Chen, Min Kang, Yan Li, Yonghui He, Zefeng Yang, Meng Zhang

**Affiliations:** 1 Institute of Infectious Disease Control and Prevention, Guangdong Provincial Center for Disease Control and Prevention, Guangzhou, Guangdong, China; 2 Hubei Provincial Center for Disease Control and Prevention, Wuhan, Hubei, China; 3 China Field Epidemiology Training Program, Beijing, China; 4 Center for Disease Control and Prevention of Sanshui, Foshan, Guangdong, China; 5 Center for Disease Control and Prevention of Foshan, Foshan, Guangdong, China; 6 National Key Laboratory of Intelligent Tracking and Forecasting for Infectious Diseases, Chinese Center for Disease Control and Prevention, Beijing, China; University of Dhaka, BANGLADESH

## Abstract

**Background:**

In March 2025, Guangdong Province reported China’s earliest locally acquired dengue case, occurring two months before the typical transmission season. This unprecedented event raised critical questions about potential overwintering transmission mechanisms in subtropical regions. The investigation sought to determine whether this case represented a resurgence from the 2024 outbreak or a new importation event.

**Methods:**

We conducted comprehensive epidemiological, entomological, and laboratory investigations to trace the origin of infection. Standardized case interviews were conducted to record the patient’s 14-day exposure history, including travel patterns. A targeted investigation within a 100-meter radius of the case exposure sites was conducted. Active surveillance and entomological assessments—including Breteau Index calculations and ovitrap deployment—were implemented within 200-meter radius of exposure sites. Serum samples were tested for DENV using real-time RT-PCR, NS1 antigen, and IgM/IgG antibodies. The envelope (E) gene was sequenced using Sanger sequencing, followed by phylogenetic analysis against global variants with the Neighbor-Joining method in MEGA software.

**Principal Findings:**

Genomic analysis of the envelope (E) gene revealed 99.93% identity between the case’s dengue virus serotype 1 strain and a locally acquired 2024 case, differing by only a single-nucleotide variant. The high-risk area showed a relatively high dengue prevalence within the previous outbreak year. Meteorological data confirmed that 22 of 26 days during the exposure window met temperature thresholds (20–25°C) conducive to mosquito activity.

**Conclusions:**

This investigation provides genomic and epidemiological evidence suggesting overwintering dengue transmission in China. The findings are consistent with local persistence from the 2024 outbreak, with vertical transmission in overwintering mosquitoes as a leading hypothesis.. These results challenge traditional assumptions about seasonal transmission patterns and underscore the need for year-round vector surveillance in subtropical regions experiencing climate warming.

## Author summary

We report genomic and epidemiological evidence suggesting overwintering dengue virus transmission in China. In March 2025, a dengue case was detected in Guangdong Province—two months earlier than the typical transmission season. Through genomic sequencing, we found that the virus was nearly identical to a strain from a local case in 2024, consistent with local persistence rather than recent importation. Our investigation showed that warm temperatures during the 2024–2025 winter allowed mosquitoes to remain active, enabling the virus to survive. Although no virus was detected in mosquitoes during our field survey, the genetic and environmental evidence suggests that the infection likely originated from locally overwintering mosquitoes, a hypothesis supported by our findings. This finding challenges the traditional view that dengue transmission in subtropical regions like Guangdong is strictly seasonal. It underscores the need for year-round mosquito surveillance and control, especially as global temperatures rise. Our study serves as an early warning for public health systems to adapt to changing climate conditions and prevent unexpected outbreaks outside the typical dengue season.

## Introduction

Dengue fever, an acute infectious disease primarily transmitted by *Aedes* mosquitoes, is endemic in more than 100 countries in tropical and subtropical regions. Reported cases reached 5.2 million worldwide in 2019, with reports surpassing 6.5 million in 2023 [1]. In China, dengue fever is predominantly distributed across southern regions, where Guangdong Province ranks among the most affected areas [2,3]. The sole vector for dengue transmission in Guangdong is *Aedes albopictus* [4,5]. Transmission typically occurs seasonally from May to December, a pattern driven by the mosquito’s ecology. During winter and early spring, mosquito populations decline due to low temperatures, naturally interrupting the virus transmission cycle and making persistent overwintering of the virus a rare event [6,7].

On March 24, 2025, Foshan City in Guangdong Province, China, reported the country’s first locally acquired dengue case of the year. This case, with the earliest onset date recorded since 2005, occurred over two months before the typical transmission season, presenting a significant epidemiological anomaly. This exceptionally early case challenged the conventional understanding of dengue virus seasonality and pointed to the potential for overwintering transmission—the persistence of dengue virus through winter leading to autochthonous cases before the normal transmission season. Therefore, we conducted this investigation to trace the origin of this exceptionally early infection. The findings aim to address a critical knowledge gap regarding the potential for and mechanisms of dengue virus overwintering in subtropical regions like Guangdong, which has significant implications for refining early warning systems and optimizing the timing of public health interventions.

## Methods

### Ethics approval and patient consent

The study was approved by the Institutional Review Board of the Guangdong Provincial Center for Disease Control and Prevention (Guangdong CDC), and written informed consent was obtained from the index case patient. The household-based epidemiological survey did not involve any child participants. The youngest participant in the study was 19 years of age, meeting the criteria of inclusion as an adult. The study protocol ensured full anonymization of all participants, and no personally identifiable information was collected or used at any stage.

### Study design

**Fig 1:**
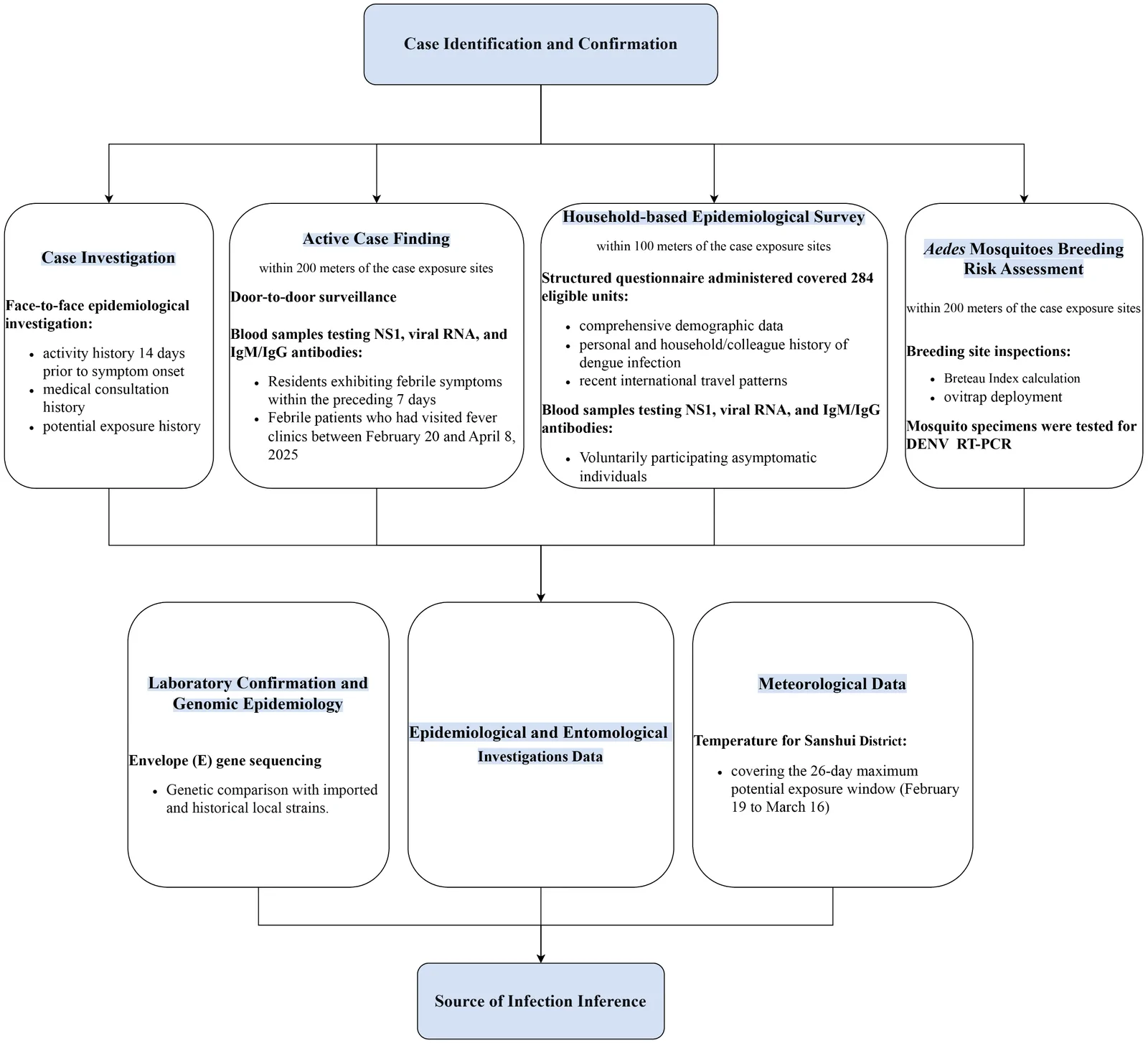
Research Design and Analytical Workflow.

This investigation employed a comprehensive, field-based approach to trace the origin of the index case. The study integrated multiple methodological components—epidemiological, entomological, meteorological, and laboratory analyses—to assess the possibility of overwintering transmission. The overall study design and workflow are visually summarized in Fig 1, which outlines the step-by-step process from case identification to data integration. Notably, the 200-meter and 100-meter radii for active surveillance and the household survey were selected based on distinct considerations: the former follows national guidelines for vector control coverage, while the latter was chosen to ensure data quality under logistical constraints.

### Case investigation

We conducted a face-to-face epidemiological investigation, which involved direct interview with the patient to gather detailed information on activity history 14 days prior to symptom onset, medical consultation history, and potential exposure history. This direct investigation served as the foundational step of the epidemiological investigation protocol [8], crucially informing the delineation of high-risk areas and guiding all subsequent field activities, including the household survey and entomological assessment.

### Active case finding

High-risk areas were defined as the case’s residence, workplace, and their 200-meter buffer zones, in accordance with the National Technical Guide for Dengue Fever Prevention and Control [9] to cover the typical flight range of *Aedes* vectors. Door-to-door surveillance was conducted for residents and businesses within this radius. Blood samples were collected from residents exhibiting febrile symptoms within the preceding 7 days. Additionally, archived serum specimens from febrile patients who had visited fever clinics in the high-risk areas between February 20 and April 8, 2025 were obtained for testing. All collected samples underwent comprehensive laboratory analysis, including testing for dengue virus (DENV) Nonstructural Protein 1 Antigen (NS1), viral RNA, and IgM/IgG antibodies.

### Household-based epidemiological survey

From March 31 to April 3, 2025, we conducted a targeted investigation within a 100-meter radius of the case exposure sites. This radius was selected to ensure high-quality data collection under operational constraints. A structured questionnaire (S1 File) was administered to one adult representative from each of the 284 eligible households and businesses, collecting demographic data, personal or household/colleague history of dengue infection, and recent international travel patterns. The collection of this sociodemographic information was for the purpose of characterizing the population at risk and contextualizing the exposure findings. Blood samples were collected from voluntarily participating residents.

### *Aedes* mosquitoes breeding risk assessment

From March 31 to April 3, 2025, we conducted systematic *Aedes* mosquitoes surveillance within 200-meter of the case’s residence and workplace. Field investigators surveyed all accessible indoor and outdoor environments within each high-risk area. At each location, potential breeding containers were recorded and categorized (e.g., indoor plant saucers, outdoor water storage tanks, discarded tires, drainage ditches, tree holes, construction debris). All water containers positive for mosquito immatures were sampled, and collected larvae were sent to the Guangdong CDC laboratory for DENV real‑time RT‑PCR testing.

Concurrently, a team deployed 13 standard ovitraps across the exposure sites, following national guidelines for vector surveillance. The traps were placed in shaded locations, and were retrieved after seven days. Retrieved egg papers were examined under a stereomicroscope, eggs were counted, and the New Ovitrap Index was calculated as the number of eggs per trap per day. All collected egg batches were tested for DENV real‑time RT‑PCR testing.

### Meteorological data

According to WHO definitions of dengue fever incubation periods - intrinsic incubation period (IIP: 1–14 days in humans) and extrinsic incubation period (EIP: 8–12 days in mosquitoes) - we retrieved temperature data for Sanshui District from the China Meteorological Administration website covering the 26-day maximum potential exposure window (February 19 to March 16), calculated as the sum of maximum IIP and EIP durations prior to the case’s symptom onset. Temperature was selected as the focal meteorological variable because it is the principal limiting factor for mosquito activity and viral replication during the winter–spring transition. This focus is justified as the study area experiences a rain-scarce winter, yet the relative humidity remains within a range conducive to mosquito survival. Consequently, mosquito breeding is sustained by artificial containers, making temperature the primary limiting factor for off-season transmission.

### Statistical analysis

All statistical analyses were performed using IBM SPSS Statistics (Version 27.0). Descriptive statistics were used to summarize the demographic characteristics of the surveyed population; categorical variables were presented as frequencies and percentages (N, %). Group comparisons between participants from Leping Town and Xinan Subdistrict—including dengue diagnosis history, suspected symptoms, and international travel—were performed using Fisher’s exact test, given small expected cell counts in some contingency tables. A two-sided p-value of less than 0.05 was considered statistically significant.

### Laboratory methods for dengue virus detection and genomic analysis

Serum samples were tested for dengue virus RNA, NS1 antigen, and virus-specific IgM/IgG antibodies. Viral RNA was extracted from 200 μL of serum using the Ex-DNA/RNA Kit (Tianlong, China; Cat. No. 24041010T330). Serotype identification was performed by real-time RT-PCR using commercial dengue virus typing kits (Aodong, China; Lot numbers: A4D040 for DENV-1, A4D041 for DENV-2, A4D042 for DENV-3, and A4D043 for DENV-4). The NS1 antigen and IgM/IgG antibodies were detected using the Dengue Virus NS1 Antigen/IgM/IgG Combined Test Kit (Colloidal Gold; Innovita, Beijing, China) in accordance with the manufacturer’s instructions.

For genomic characterization, the envelope (E) gene (1,485 bp) of the RT-PCR-positive serum sample using two overlapping primer pairs designed based on conserved regions of DENV-1 sequences (Table 1). RT-PCR amplification was performed using the SuperScript III One-Step RT-PCR with Platinum Taq kit (Invitrogen, USA) with the reaction mixture shown in Table 2 Thermal cycling conditions were as follows: reverse transcription at 50°C for 30 min; initial denaturation at 94°C for 2 min; 39 cycles of 94°C for 15 s, 55°C for 30 s, and 68°C for 90 s; and a final extension at 68°C for 5 min. PCR products were purified and subjected to Sanger sequencing. Chromatograms were assembled into consensus sequences using SeqMan (DNASTAR) with manual verification.

**Table 1:** Primers used for amplification of the DENV-1 envelope (E) gene.

Primer	Sequence (5’ → 3’)	Position	Amplicon size
D1F1	CACATGCCATAGGAACATCC	858	1,028 bp
D1R1	ATGCTGGGTCTCAGCCACTT	1885	
D1F2	GGAAYAGACAAGAYTTGCTGGT	1626	849 bp
D1R2	TGCCRCTTCCACATTTGAGT	2474	

**Table 2:** RT-PCR reaction mixture for DENV-1 E gene amplification.

Component	Volume (μL)
2 × Reaction Mix	12.5
Primer D1F1/D1F2 (10 μM)	0.5
Primer D1R1/D1R2 (10 μM)	0.5
Enzyme mix	1
Template RNA	5
RNase-free water	5.5
**Total**	**25**

For phylogenetic analysis, multiple sequence alignment of the 1,485-bp envelope (E) gene sequences was performed using BioEdit (version 7.0.9.0) with manual adjustment. The phylogenetic tree was reconstructed using the Neighbor-Joining method implemented in MEGA11, with branch support evaluated by 1,000 bootstrap replicates.

All collected mosquito specimens (larvae and egg batches) were tested for DENV using real-time RT-PCR with commercial kits (BioGerm, China) following the manufacturer’s instructions. The assay was performed on a Bio-Rad C1000 Touch thermal cycler, with a cycle threshold (Ct) value < 38 considered positive for DENV RNA. The assay has a limit of detection of 500 copies/mL as specified by the manufacturer. All mosquito specimens tested negative; therefore, no Ct values are reported.

## Results

### Case investigation

On March 24, 2025, Guangdong Province reported China’s first locally acquired dengue fever case of the year—the earliest annual onset in the province since 2005. The patient, a 50-year-old female accountant from Foshan City, developed fever on March 17 without significant respiratory symptoms. After seeking care at multiple hospitals from March 18–21 with no improvement, she was tested on March 22, revealing leukopenia, thrombocytopenia, and a positive NS1 antigen. Admitted under mosquito-proof isolation as a suspected dengue case, she was later confirmed to have dengue virus serotype 1 (DENV-1) infection, with weakly positive IgM antibodies.

During the 14-day incubation period (March 3–16), the patient primarily stayed within Sanshui District, except for a brief visit to Chancheng District on March 4 (both in Foshan City). Her routine consisted of daily bus commutes between her primary residence (Leping Town) and workplace (Xinan Subdistrict), with midday breaks at her parents’ residence (Xinan Subdistrict) located approximately 600m from her workplace. Notably, from March 10–14, she remained continuously at her parents’ residence without returning to her own home. Weekend activities were limited to the area around the residence. Additionally, she reported mosquito bites at her parents’ residence during this period.

### Active case finding

Investigations of the case’s husband, parents-in-law, parents, and 7 workplace colleagues revealed no fever. Door-to-door screening within the 200-meter radius of the workplace and residences, covered all 2,919 households (7,890 individuals). During active surveillance, 12 febrile cases (with fever onset within prior 7 days) were identified and sampled. We also retrieved 64 archived serum samples from febrile patients treated at four healthcare facilities near the affected areas (February 19-April 8). All specimens tested negative for DENV NS1 antigen, viral RNA, and IgM/IgG antibodies through standardized testing protocols.

### Household-based epidemiological survey

We conducted an epidemiological survey within a 100-meter radius of the exposure sites, covering 284 eligible households and businesses. The survey included 284 participants with a male-to-female ratio of 0.69:1 (116 males, 168 females). The age of participants ranged from 26 to 74 years, with 51.78% between 30 and 49 years. The majority (48.24%) of participants worked in commercial or service sectors, followed by retirees (22.89%), and homemakers or unemployed individuals (10.56%). Regarding education, 51.41% of participants had completed Secondary education (Junior high school/ General High school/ Secondary vocational school). Full demographics are shown in Table 3. These demographic characteristics indicate that the surveyed population was broadly representative of the adult residents in the two communities, supporting the generalizability of the exposure and infection history findings. A total of 9 blood samples were collected from residents—7 from Leping Town and 2 from Xinan Subdistrict. All samples tested negative for dengue virus NS1 antigen, viral RNA, and IgM/IgG antibodies.

**Table 3:** Demographic Characteristics of Surveyed Population.

Category	Leping Town		Xinan Subdistrict		Total	
	N	*%*	N	*%*	N	*%*
**Sex**						
Male	10	62.50	106	39.55	116	40.85
Female	6	37.50	162	60.45	168	59.15
**Age (years)**						
10-19	0	0.00	1	0.37	1	0.35
20-29	1	6.25	15	5.60	16	5.63
30-39	2	12.50	79	29.48	81	28.52
40-49	3	18.75	63	23.51	66	23.24
50-59	2	12.50	39	14.55	41	14.44
60-69	4	25.00	43	16.04	47	16.55
70-79	4	25.00	15	5.60	19	6.69
80-	0	0.00	13	4.85	13	4.58
**Occupation**						
Commerce/Service	4	25.00	133	49.63	137	48.24
Retirees	6	37.50	59	22.01	65	22.89
Homemaker/Unemployed	4	25.00	26	9.70	30	10.56
Office Worker	0	0.00	17	6.34	17	5.99
Laborer/Migrant Worker	2	12.50	11	4.10	13	4.58
Food Service	0	0.00	10	3.73	10	3.52
Healthcare Worker	0	0.00	5	1.87	5	1.76
Teacher	0	0.00	3	1.12	3	1.06
Other	0	0.00	4	1.49	4	1.41
**Education**						
Primary school or below	6	37.50	45	16.79	51	17.96
Secondary education (Junior high school/ General High school/ Secondary vocational school)	8	50.00	138	51.49	146	51.41
Higher Vocational College	0	0.00	48	17.91	48	16.90
University (bachelor’s degree) or above	2	12.50	37	13.81	39	13.73

Among 268 participants near the index case’s exposure sites in Xinan Subdistrict, 22 (8.2%) had laboratory-confirmed dengue fever in 2024, with 8.0% (17/212) reporting cohabitants or colleagues who were also diagnosed. Analysis of these 22 cases showed that 6 cases lived within 50 m of the index case’s residence or workplace; of these, two were near her workplace and four were near her residence. The remaining 16 cases lived within a 50–100-meter radius of these locations. Notably, 27.3% (6/22) had cohabitants or colleagues with dengue diagnoses, while 77.3% (17/22) reported dengue-like symptoms. Regarding travel history, only 9 participants and 3 close contacts had visited Hong Kong/Macao since February 2025, with no travel to dengue-endemic regions (Southeast Asia, Africa, or South America). All surveyed participants from Leping Town and their cohabitants/colleagues reported no history of dengue fever diagnosis nor overseas travel (Table 4). The complete anonymized dataset underlying the household-based survey, including all demographic variables and individual responses, is provided in S1 Table.

**Table 4:** Dengue Infection and Overseas Travel History Among Residents in High-risk Areas.

Exposure History	Total (N)	%	Leping Town (N)	Xinan Subdistrict (N)	P-value^a^
**Diagnosed with dengue since May 2024**					**0.622**
Yes	22	7.75	0	22	
No	262	92.35	16	246	
**Suspected dengue symptoms since May 2024**					**0.377**
Yes	26	9.15	0	26	
No	258	90.85	16	242	
**Household/colleague diagnosed since May 2024**					**0.815**
Yes	17	7.56	0	17	
No	205	92.44	13	192	
**International travel since February 2025**					**1.000**
Yes	9	3.17	0	9	
No	275	96.83	16	259	
**Household/colleague traveled since February 2025**					**1.000**
Yes	3	1.33	0	3	
No	222	98.67	13	209	

^a^Fisher’s Exact Test.

A summary of all laboratory testing performed on nearby human populations, stratified by symptom status, is presented in Table 5. Apart from the index case, all symptomatic and asymptomatic individuals tested negative for DENV, indicating no evidence of undetected transmission among nearby residents during the study period.

**Table 5:** Summary of laboratory testing results in nearby human populations.

Population group	Symptom status	N	Assays performed	Sampling period	Timing relative to exposure window	Results
Febrile residents (active case finding)	Symptomatic	12	RT-PCR, NS1 antigen, IgM/IgG	March 24– 29	8–13 days after exposure window	All negative
Febrile patients (archived sera, nearby clinics)	Symptomatic	64	RT-PCR, NS1 antigen, IgM/IgG	Feb 19–April 8	During and after exposure window	All negative
Household survey volunteers	Asymptomatic	9	RT-PCR, NS1 antigen, IgM/IgG	March 31–April 3	15–18 days after exposure window	All negative

### *Aedes* mosquitoes breeding risk assessment

An assessment of the exposure sites revealed distinct environmental profiles conducive to mosquito breeding. The primary case’s exposure site in Xinan Subdistrict was located in an old urban area characterized by high population density, aging residential complexes, and diverse mosquito breeding habitats. The Leping Town exposure site represented a rural setting, with predominant breeding sites including water containers in peri-domestic environments, vegetable plots, and abandoned land.

Entomological surveys collected a total of 13 *Aedes* larvae and 8 *Aedes* egg batches, all exclusively from the Xinan Subdistrict sites. Detailed collection data revealed that all 13 larvae were obtained from within 50 m of the residence. The 8 egg batches were collected from two areas: 3 from within 50 m of the workplace and 5 from within 100-meter of the secondary residence (her parents’ resident). In contrast, no larvae, eggs, or active breeding sites were found at the Leping Town exposure site. Morphological identification confirmed that all collected specimens belonged to the genus *Aedes*. All collected specimens tested negative for DENV by RT-PCR.

### Spatial distribution of sampling and laboratory findings

**Fig 2:**
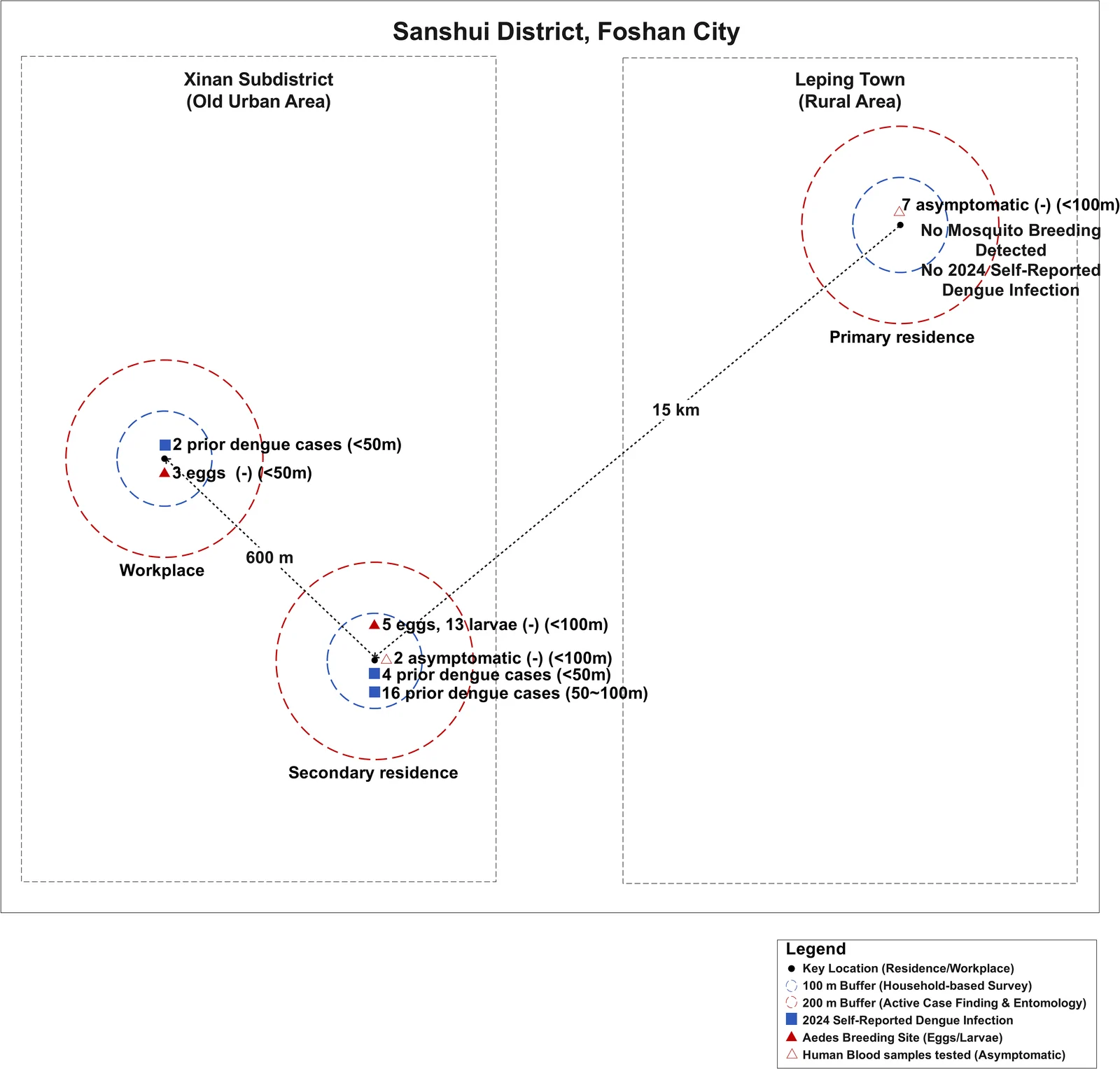
Spatial Distribution of Sampling and Laboratory Findings in Sanshui District, Foshan City.

The geographic relationships between the two main exposure sites, the distribution of 2024 self-reported dengue cases, and the results of human and mosquito sampling are summarized in Fig 2. Key findings presented in the map include the spatial separation of Leping Town and Xinan Subdistrict, the location of 22 self-reported 2024 cases within 100-meter of the Xinan Subdistrict exposure sites, and the collection sites of 13 *Aedes* larvae and 8 *Aedes* egg batches. All human blood samples and mosquito specimens collected from both sites tested negative for DENV, as detailed in the preceding sections.

(A) Relative positions of Leping Town (rural area) and Xinan Subdistrict (old urban area), separated by approximately 15 km. (B) Xinan Subdistrict detail: generalized 200-m (red dashed line) and 100-m (blue dashed line) buffer zones around the workplace and Secondary residence (black circles). Approximate locations of 2024 self-reported dengue infections (n = 22) are indicated within the 100-m zone. Mosquito sampling identified *Aedes* breeding sites (red solid triangles) within the buffer zones. (C) Leping Town detail: Residence 2 (black circle) with identical buffer zones; no mosquito breeding sites were detected. Sampling locations for asymptomatic volunteers (red hollow triangles) are shown. The 12 febrile residents identified through active case finding and the 64 archived sera from febrile patients are not individually mapped due to the absence of precise location data for these samples. All human and mosquito samples collected across both regions tested DENV-negative. All precise geographic coordinates were obfuscated to protect case confidentiality.

### Meteorological data

**Fig 3:**
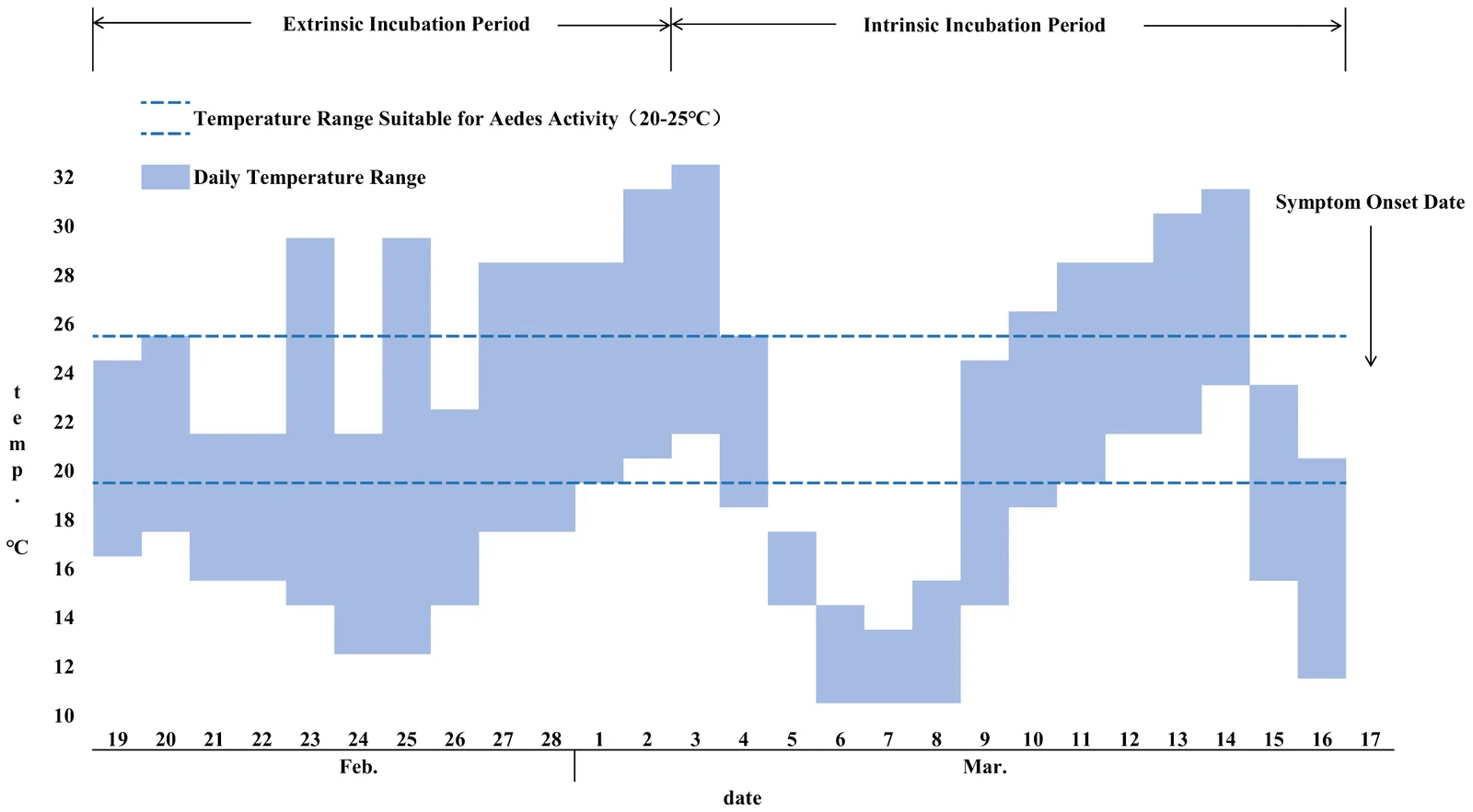
Temperature Fluctuations Relative to Dengue Virus Incubation Periods in Sanshui District (February 19 - March 16).

Sanshui District experienced warmer-than-average temperatures during the 2024–2025 winter (December-February), with detailed analysis showing that 22 of the 26 days preceding the case’s symptom onset (February 19 to March 16) reached temperatures conducive to mosquito activity (20–25°C). During the extrinsic incubation period (February 19-March 2), all 12 days met this thermal threshold, while 10 of 14 days in the intrinsic incubation period (March 3–16) fell within the active temperature range for mosquito transmission (Fig 3). The daily temperature records used in this analysis are provided in S2 Table.

### Laboratory confirmation and genomic epidemiology

**Fig 4:**
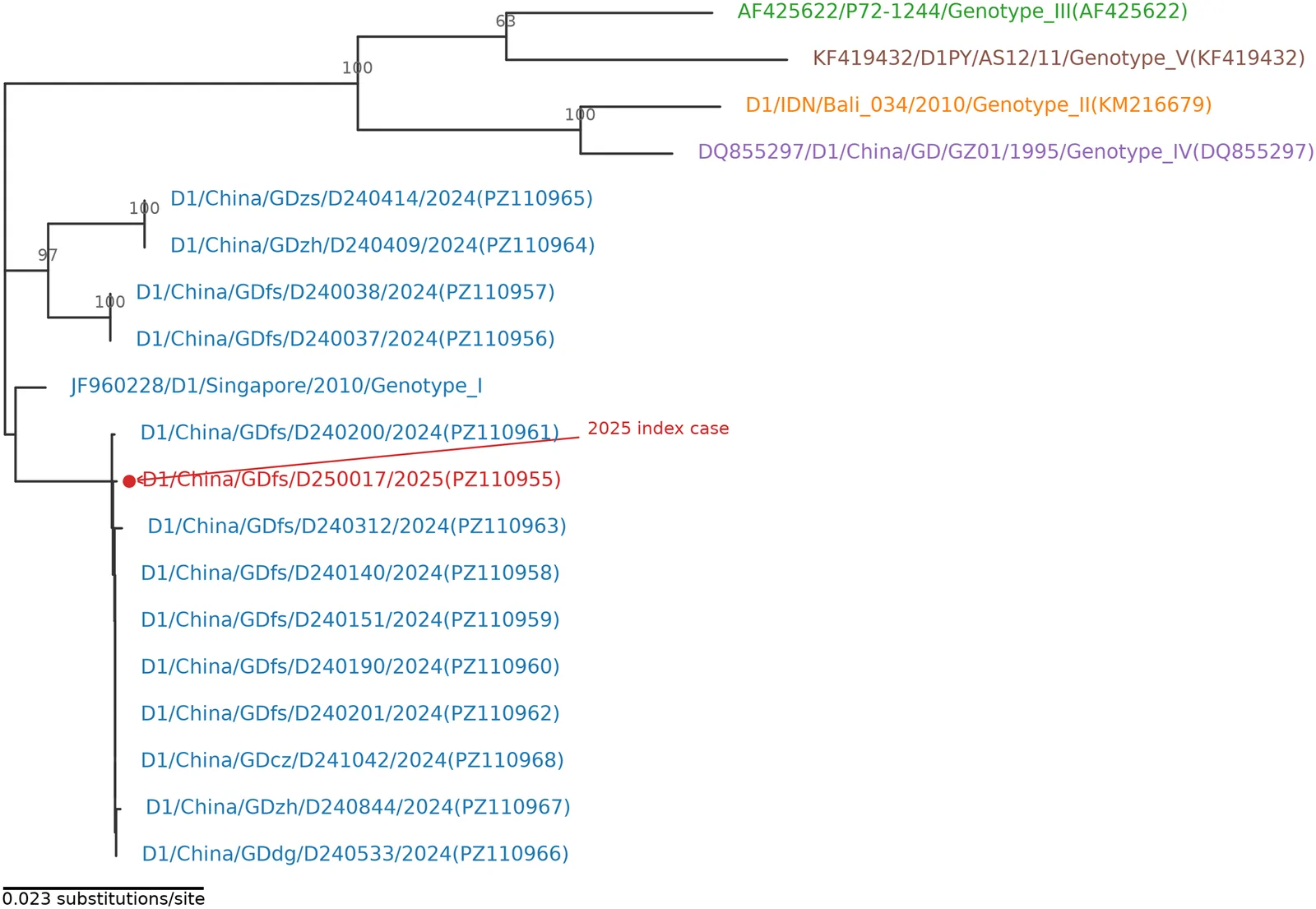
Phylogenetic Tree of E Gene of the Dengue Index Case in Guangdong Province in 2025.

The Guangdong CDC performed sequencing of the envelope (E) gene on March 25. Phylogenetic analysis identified the virus as DENV-1 genotype I, revealing 99.93% genetic similarity with a locally acquired DENV-1 case from Xinan Subdistrict in 2024, differing by only a single-nucleotide variant. The 2024 case resided approximately 1.8 km from both the residence and workplace of the current case. Phylogenetic analysis against global databases demonstrated no higher similarity matches, with the closest being strain 14906/D1/Selangor/2019 from Singapore (98.99% similarity, uploaded January 2024) (Fig 4). The multiple sequence alignment file and the phylogenetic tree file are provided as S2 and S3 Files.

The Neighbor-Joining tree was reconstructed based on 1,485-bp E gene sequences, with 1,000 bootstrap replicates. The scale bar represents nucleotide substitutions per site. The 2025 index case sequence is indicated by a red dot (PZ110955). Reference sequences from GenBank are labeled with their strain names and accession numbers.

### Outcome evaluation

The public health response to China’s first locally acquired dengue case of 2025, successfully achieved transmission interruption. Through 25 days of enhanced surveillance and vector control, no secondary cases were identified. Mosquito density monitoring demonstrated sustained suppression below the safety threshold (Breteau Index <5) from March 30 onward. Combined epidemiological and entomological evidence confirms this constituted an effectively contained sporadic case without community transmission.

## Discussion

Based on epidemiological investigation and laboratory testing results, this case was confirmed as a locally acquired dengue fever infection. The case likely represents a continuation of the 2024 local dengue outbreak, with possible infection resulting from a bite by virus-carrying overwintering mosquitoes. Key evidence includes: 1) The case had no history of international travel or travel outside Foshan City within 14 days prior to symptom onset. 2) Envelope (E) gene sequencing revealed >99.9% sequence identity between this strain and a DENV-1 strain from another 2024 case in Xinan Subdistrict, where the patient worked and lived. 3) Field investigation found that 22 of the 268 respondents within 100m of the the index case’s exposure sites reported being diagnosed with dengue fever in the past year. 4) None of the surveyed participants near these exposure sites reported recent travel to dengue-endemic regions (Southeast Asia, South America, or Africa). 5) Relatively warm temperatures during the 2024–2025 winter-spring period facilitated *Aedes* mosquitoes overwintering. During the case’s potential exposure window (February 19 to March 16), daily mean temperatures in Sanshui District remained at 20–25°C. Further field investigations not only identified active mosquito breeding sites but also detected larval presence, thereby confirming sustained mosquito activity under these climatic conditions.

Vertical transmission occurs either via transovarial transmission (viral infection of female germline tissues) or through trans-ovum transmission, the latter encompassing both fertilization-stage infection and post-maturation egg infection during oviposition [10–12]. In the laboratory, vertical transmission may be confirmed by detecting the virus in the offspring of orally or intrathoracically inoculated females [12,13], whereas in nature it is assumed when the virus is detected in the immature mosquito stages or male mosquitoes [14]. Both experimental [11,15–17] and field studies [18–20] across multiple countries have consistently demonstrated that *Aedes* mosquitoes serve as natural reservoirs for DENV. Particularly, a study in Guangzhou (adjacent to Foshan City) also confirmed this conclusion [6]. Furthermore, research from Taiwan indicates that overwintering DENV-infected mosquitoes may undergo viral genetic or molecular adaptations, potentially shaping outbreak intensity and epidemiological patterns in subsequent years [21]. Based on these foundational research findings, vertical transmission in mosquito vectors is widely regarded as a mechanism for environmental viral persistence and epidemic amplification. However, direct epidemiological evidence remains limited. As such, our work contributes valuable field-based evidence supporting this hypothesized linkage.

Several alternative explanations for this early case warrant consideration: 1) Cryptic low-level transmission: Unusually warm temperatures could sustain undetected transmission, but no DENV infections were identified among 12 febrile residents, 64 archived sera from febrile patients, and 9 asymptomatic volunteers. No cases were found in Xinan Subdistrict in the subsequent month, arguing against this possibility. 2) Importation with missed travel linkage: The case and nearby populations denied international travel, and the strain is > 99.9% identical to a 2024 local case, not imported strains. 3) Transmission from outside the surveyed radius: Infected mosquitoes beyond 200-meter cannot be ruled out. However, enhanced febrile surveillance was implemented across Sanshui District following the case report, requiring dengue testing for all febrile patients presenting at healthcare facilities. No confirmed DENV infections were identified beyond the surveyed area during this surveillance period, arguing against significant transmission from outside the high-risk areas. 4) Delayed sampling masking infected vectors: Sampling was delayed one week; while all specimens (13 larvae and 8 egg batches) tested negative, low field infection rates documented elsewhere indicate that negative entomological findings do not preclude the prior presence of infected vectors. For example, a study in Yogyakarta, Indonesia detected dengue virus in only 36 of 29,252 female mosquitoes (0.12%) [22], and during Egypt’s 2017 outbreak, the virus was identified in merely one pooled mosquito sample [23]. These extremely low detection rates, even during active outbreaks, suggest that infected vectors could have been present earlier but escaped detection, particularly when sampling is delayed.

This study has several limitations. First, entomological sampling was delayed by one week due to the priority implementation of vector control measures following case identification, and the number of collected specimens was limited by the availability of positive breeding sites at the time of sampling. Second, despite comprehensive breeding site inspections across the three high-risk areas, the modest sample size constrains the generalizability of the entomological findings. Third, the presence of asymptomatic or mildly symptomatic DENV infections [24] means that undetected transmission cannot be definitively ruled out, especially given Foshan’s frequent international exchanges and the persistently high global dengue incidence. These limitations highlight the need for enhanced vector surveillance and rapid response protocols to enable earlier and more extensive entomological sampling in future investigations.

Despite the occurrence of overwintering mosquito-borne cases in Guangdong, current evidence remains insufficient to support the endemicity of dengue fever in this region. Currently, there are no globally standardized criteria for defining dengue localization. It is proposed that potential indicators include: year-round elevated temperatures enabling continuous *Aedes* mosquitoes activity and local case reporting throughout the year; a high proportion of cases occurring in children under 15 years old; co-circulation of all four dengue virus serotypes (DENV-1–4) with varying intensity and alternating predominance across regions and years; and a high severe disease and mortality rate, primarily affecting children. Comparative analysis of Guangdong’s epidemic characteristics with those of endemic countries in Southeast Asia and South America reveals significant differences. Guangdong typically reports local cases from May-June, occasionally starting as early as March-April (as in 2010, 2015, and 2025), continuing until December, with no cases reported in January-February. Cases predominantly affect young adults aged 20–59 years (accounting for 70%), and local transmission each year is generally dominated by a single serotype, either DENV-1 or DENV-2. In recent years, DENV-1 has been the predominant serotype in Guangdong, with genotype I being the most prevalent lineage within this serotype [25]. Furthermore, the level of severe disease and mortality is low; since 2010, severe cases in Guangdong have mostly been zero, with only a few years recording single-digit numbers. This comparative analysis indicates that dengue has not yet localized in Guangdong [26]. Consequently, the dengue epidemic pattern in Guangdong remains characterized by annual local transmission initiated by imported cases. Although the current local case raises the possibility of overwintering transmission, it does not align with the established characteristics of localization. However, given the persistent increase in global temperatures coupled with the ongoing rise in international travel to and from the province, there exists a risk of gradual dengue localization occurring in specific areas of Guangdong, necessitating heightened vigilance and proactive preparedness measures.

This study yields pivotal insights with potential implications for dengue control strategizing. These findings serve as a salient warning that local transmission risks persist even during traditional non-epidemic seasons, underscoring the importance of robust outbreak control measures during large-scale epidemics, as well as enhanced vector surveillance and early intervention to prevent resurgence in subsequent years [27]. Given the global warming trend and increasing international personnel exchanges, there is a risk of gradual dengue fever endemicity in some local areas, which requires high vigilance and proactive preparedness.

## Conclusion

This investigation provides genomic and epidemiological evidence consistent with local overwintering transmission of dengue virus in Guangdong, China. The index case’s virus shares >99.9% nucleotide identity with a 2024 local strain, and no travel history to endemic regions was identified—demonstrating that the infection likely originated from persistent local circulation rather than recent importation. Based on supporting evidence, including warm winter temperatures conducive to mosquito activity and a high density of prior cases near the exposure sites, we hypothesize that transmission occurred via overwintering infected *Aedes* mosquitoes. However, direct detection of DENV in overwintering vectors was not achieved due to sampling limitations, and alternative explanations such as cryptic low-level transmission cannot be definitively excluded. These findings challenge traditional assumptions about seasonal transmission patterns and underscore the need for year-round vector surveillance in subtropical regions experiencing climate warming.

## Supporting information

S1 Table Data of Household-based Epidemiological Survey. This file contains the complete, anonymized dataset underlying the results of the household survey, including participant demographics, history of dengue infection, and international travel records.
(XLSX)

S2 Table Daily temperature records from Sanshui District. This dataset provides the daily mean temperature for the 26-day potential exposure window (February 19 to March 16, 2025) used in the meteorological analysis.
(XLSX)

S1 File Household-based Epidemiological Survey Form. A copy of the structured questionnaire (English version) administered to adult representatives from all eligible households and businesses within the 100-meter survey radius.
(DOCX)

S2 File Multiple sequence alignment of DENV-1 E gene sequences. This file contains the aligned 1,485-bp envelope (E) gene sequences used for phylogenetic analysis, including the 2025 index case (PZ110955), and reference sequences representing the major genotypes of DENV-1. The alignment was performed using BioEdit (version 7.0.9.0) with manual adjustment.
(FAS)

S3 File Phylogenetic tree file. This file contains the Neighbor-Joining tree in Newick format generated from the E gene alignment, with 1,000 bootstrap replicates.
(NWK)
