# Supplementary material for: Genomic and epidemiological evidence linking a pre-season dengue case to local overwintering transmission in Guangdong, China
Source: PLoS Negl Trop Dis. 2026 Aug 21;20(8):e0014221. doi: 10.1371/journal.pntd.0014221 (PMC13497276; doi:10.1371/journal.pntd.0014221)
Supplement: Supplementary file 1 [file pntd.0014221.s003.docx]

Dengue Fever Household-based Epidemiological Survey Form

Hello Residents! To understand people's dengue fever infection status and better implement prevention and control measures, we are conducting a survey on dengue fever risk factors. It will take you only 2 minutes. Thank you for your cooperation.

1. Survey Household Number: ____ [Fill in the blank] *

2. Detailed Household Address: ____ Sub-district/Township, ____ Community/Village, ____ No., S District, Foshan City [Fill in the blank] *

4. Your Gender: [Single choice] *

□ Male □ Female

5. Your Age (in years): [Fill in the blank] *

6. Your Contact Phone Number: [Fill in the blank] *

7. Your Occupation: [Single choice] *

□ Catering and Food Industry Workers

□ Commercial/Service Industry Workers

□ Medical Staff

□ Logistics and Distribution Industry Workers

□ Workers/Migrant Workers

□ Cadres and Staff

□ Agriculture, Forestry, Animal Husbandry, and Fisheries Workers

□ Retirees

□ Housewives and Unemployed Persons

□ Students

□ Teachers

□ Others _________________ *

8. Educational Background: [Single choice] *

□ Primary School or Below

□ Secondary education (Junior high school/ General High school/ Secondary vocational school)

□ Higher Vocational College

□ University (bachelor's degree) or above

9. Have you been a permanent resident of Foshan since May 1, 2024? [Single choice] *

□ Yes □ No

10. Have you been diagnosed with dengue fever since May 1, 2024? [Single choice] *

□ Yes □ No

11. Have you received the yellow fever vaccine? [Single choice] *

□ Yes □ No

12. Have you experienced any of the following symptoms/signs since May 1, 2024? [Multiple choice] *

□ Fever □ Headache □ Fatigue □ Generalized Pain □ Sore Throat □ Nasal Congestion□ Sneezing □ Cough □ Orbital Pain □ Nausea □ Vomiting □ Flushing of the Face, Neck, and Chest□ Rash or Ecchymosis □ Other Symptoms _________________*

□ No Symptoms

13. Date of Onset of Symptoms (if multiple episodes, fill in the date of the most recent one): ____[Fill in the blank] *

14. How long did your symptoms last? ____ days [Fill in the blank] *

15. Did you seek medical treatment after the symptoms appeared? [Single choice] *

□ Yes □ No (Please skip to Question 17)

17. Have you had any overseas travel history since February 2025? [Single choice] *

□ Yes □ No (Please skip to Question 21)

18. Your Travel Destination (if multiple destinations, fill in the order of priority: overseas first, then the most recent one; the same below): [Fill in the blank] *

19. Start Date of Your Travel: [Fill in the blank] *

20. Duration of Your Travel: ___ days [Fill in the blank] *

21. Do you have any cohabitants? [Single choice] *

□ Yes □ No (Please skip to the end of the questionnaire and submit it)

22. Has any of your cohabitants been diagnosed with dengue fever since May 1, 2024? [Single choice] *

□ Yes □ No

23. Has any of your cohabitants had any overseas travel history since February 2025? [Single choice] *

□ Yes □ No (Please skip to Question 25)

24. Please provide their name, contact phone number, travel destination, travel duration and other relevant information:____ [Fill in the blank] *

25. Number of Permanent Residents in Your Household:____ [Fill in the blank] *
